# Phthalate concentrations in house dust in relation to autism spectrum disorder and developmental delay in the CHildhood Autism Risks from Genetics and the Environment (CHARGE) study

**DOI:** 10.1186/s12940-015-0024-9

**Published:** 2015-06-26

**Authors:** Claire Philippat, Deborah H. Bennett, Paula Krakowiak, Melissa Rose, Hyun-Min Hwang, Irva Hertz-Picciotto

**Affiliations:** Divisions of Epidemiology and of Environmental and Occupational Health, Department of Public Health Sciences, School of Medicine, University of California, Davis, CA USA; MIND Institute, University of California, 2825 50th Street, Sacramento, CA 95817 USA; Department of Civil and Environmental Engineering, University of California, Davis, CA USA; Department of Environmental and Interdisciplinary Sciences, Texas Southern University, Houston, TX USA

**Keywords:** Autism spectrum disorder, Developmental delay, Home dust, Phthalates

## Abstract

**Background:**

Phthalates are endocrine-disrupting chemicals that influence thyroid hormones and sex steroids, both critical for brain development.

**Aim:**

We studied phthalate concentrations in house dust in relation to the risks of developing autism spectrum disorder (ASD) or developmental delay (DD).

**Methods:**

Participants were a subset of children from the CHARGE (*CH*ildhood *A*utism *R*isks from *G*enetics and the *E*nvironment) case–control study. ASD and DD cases were identified through the California Department of Developmental Services system or referrals; general population controls were randomly sampled from state birth files and frequency-matched on age, sex, and broad geographic region to ASD cases. All children (50 ASD, 27 DD, 68 typically developing (TD)) were assessed with Mullen Scales of Early Learning, Vineland Adaptive Behavior Scales (VABS) and Aberrant Behavior Checklist. We measured 5 phthalates in dust collected in the child’s home using a high volume small surface sampler.

**Results:**

None of the phthalates measured in dust was associated with ASD. After adjustment, we observed greater di(2-ethylhexyl) phthalate (DEHP) and butylbenzyl phthalate (BBzP) concentrations in indoor dust from homes of DD children: Odds ratios (OR) were 2.10 (95 % confidence interval (CI); 1.10; 4.09) and 1.40 (95 % CI; 0.97; 2.04) for a one-unit increase in the ln-transformed DEHP and BBzP concentrations, respectively. Among TD children, VABS communication, daily living, and adaptive composite standard scores were lower, in association with increased diethyl phthalate (DEP) concentrations in dust. Participants with higher dibutyl phthalate (DBP) concentrations in house dust also trended toward reduced performance on these subscales. Among ASD and DD boys, higher indoor dust concentrations of DEP and DBP were associated with greater hyperactivity-impulsivity and inattention.

**Discussion and conclusion:**

House dust levels of phthalates were not associated with ASD. The inability to distinguish past from recent exposures in house dust and the fact that house dust does not capture exposure from all sources, limit the interpretation of both positive and null findings and further work is needed. However, the associations observed for DEP and DBP with impairments in several adaptive functions and greater hyperactivity, along with evidence for increased risk of DD raise concerns that these chemicals may affect neurodevelopment in children.

**Electronic supplementary material:**

The online version of this article (doi:10.1186/s12940-015-0024-9) contains supplementary material, which is available to authorized users.

## Background

Autism spectrum disorder (ASD) is a neurodevelopmental disability characterized by impairments in social interaction and communication and stereotyped behaviors [1]. Symptoms usually appear in the early developmental period and generally lead to serious lifelong limitations in functioning. The genetic component to ASD is well recognized; however, it has been suggested that an array of non-genetic factors may also play a role in the development of this disorder [2] and heritability estimates provide further support [3]. In this study, we explored the potential for neurodevelopmental effects of phthalates, a family of man-made chemicals which have recently received considerable attention because of their endocrine disrupting properties and their high level of production leading to widespread exposure in the general population [4]. Low molecular weight (MW) phthalates (MW < 250 g/mol) are primarily used in personal care products (perfumes, cosmetics) and as coatings for pharmaceutical products. High MW phthalates are mostly used in food packaging, medical devices and polyvinyl chloride (PVC) plastics found in floor and wall coverings and furniture, and are also common constituents of paints and varnishes [5]. Phthalates are not chemically bound to these materials, they can be released into indoor air, and then partition into dust [6]. In a study measuring 89 organic chemicals in indoor air and dust sampled between 1999 to 2001 from 120 American homes, phthalates were among the most abundant compounds [7].

Phthalates are endocrine disruptors and can disturb the thyroid [8, 9] and sex hormone [10] homeostasis, both critical for pre- and early post-natal brain development. They are also weak agonists of the aryl hydrocarbon receptor (AhR) [11] expressed in neural progenitor cells and human brain tissues, among others. Only a few studies have explored the associations between phthalate exposures and the risk of developing ASD in humans. One study used the Social Responsiveness Scale (SRS), a questionnaire completed by the parents and developed to detect and measure the severity of ASD behaviors, and reported greater social deficits at ages 7 to 9 in association with increased concentrations of monoethyl phthalate (MEP), a metabolite of diethyl phthalate (DEP), in maternal urine collected during pregnancy (*n* = 137 mother child-pairs from the general population [12]). Using the same instrument among younger children (4 to 5 years old), Braun et al. [13] observed a negative association (consistent with fewer ASD symptoms) with MEP concentration measured in maternal urine collected during pregnancy (*n* = 175 mother child-pairs from the general population. In a cross-sectional study focused on di(2-ethylhexyl) phthalate (DEHP), higher concentrations of DEHP metabolites have been reported among children with ASD compared to sex- and age-comparable controls (*n* = 48 children with ASD and 45 controls, [14]). The cross-sectional design is problematic because of potential reverse causality; that is, behaviors or medication taken by children with ASD may lead to higher phthalate amounts absorbed and hence higher urinary phthalate levels. Finally, an increased risk of developing ASD during childhood has been reported in association with the presence of PVC flooring in the parents’ bedroom [15].

In humans, phthalate exposures have also been associated with other aspects of child neurodevelopment including deficits in cognitive and psychomotor development, greater externalizing behaviors (e.g., aggression), and higher risk for attention-deficit/hyperactivity disorders (reviewed by Miodovnik et al. [16]).

Our aim was to examine the relationship between phthalate exposures, assessed by measuring phthalate concentrations in house dust collected 2 to 5 years after birth, and diagnosis of ASD or developmental delay (DD) in early childhood. To our knowledge, no study regarding intra-home temporal variability in phthalate dust concentrations has been published. However, temporal variability in indoor air has been previously assessed: a study conducted in New York City apartments reported moderate to low variability (intraclass correlation coefficients (ICC) were moderate to high, ranging from 0.48 for DEHP to 0.83 for butylbenzyl phthalate (BBzP)), for phthalate concentrations measured in indoor air [17]. For these reasons, we considered phthalates in house dust collected 2 to 5 years after birth as a measure of one direct source of the child’s current and longer-term exposure (e.g., during pregnancy and early postnatal life, two crucial time periods for brain development).

## Methods

### Study population

Participants were a subset of children from CHARGE (*CH*ildhood *A*utism *R*isks from *G*enetics and *E*nvironment), a population-based case–control study that aims to understand both genetic and environmental causes of ASD and DD [18]. CHARGE enrolled 2 to 5 years old children from three strata: children with ASD, children with DD without ASD, and children from the general population. Children with ASD and DD were identified through the California Regional Center system, which contracts with the State Department of Developmental Services (DDS), lists obtained from the DDS directly, referrals from other research studies at the MIND (Medical Investigations of Neurodevelopmental Disorders) Institute and from various clinics, and self-referrals. Children from the general population were randomly sampled from state birth files and frequency matched on age, sex, and broad regions of residence defined by the Regional Center catchment areas of CHARGE ASD participants. Inclusion criteria for CHARGE were the following: being between the ages of 24 and 60 months and born in California, living with at least one biologic parent who speaks English or Spanish, and residing in the catchment areas of an *a priori* specified list of Regional Centers in California. No exclusions were made based on genetics or family phenotype [18]. Children were eligible for the dust study if they (1) were enrolled in CHARGE between January 2010 and July 2011 or were enrolled in CHARGE before 2010 but were still under 60 months of age during the dust study enrollment, and (2) lived in the same home with carpeting since the child’s birth (or had retained a large area rug from that time period). Families of 167 children who met these selection criteria agreed to participate. Among them 145 had a confirmed diagnosis (see below) and were included in the present analysis.

The CHARGE study protocol has been approved by institutional review boards of the University of California in Davis and the State of California Committee for the Protection of Human Subjects. Written informed consent was obtained from each participant before enrollment.

### Outcomes

All diagnoses were confirmed using multiple standardized instruments. Trained clinicians evaluated all children enrolled with a prior diagnosis of ASD using the Autism Diagnostic Interview, Revised (ADI-R) [19] and the Autism Diagnostic Observation Schedule (ADOS) [20]. Children without ASD were assessed on the Social Communication Questionnaire (SCQ), which was developed to screen for evidence of ASD symptoms. Those who scored 15 or higher were then administered the ADI-R and ADOS.

Cognitive and adaptive functioning was assessed in all children (ASD, DD and population controls using the Mullen Scales of Early Learning (MSEL) and Vineland Adaptive Behavior Scales (VABS), respectively. MSEL consists of four subscales that measure fine motor, visual reception, expressive language, and receptive language and was administrated to children by trained clinicians [21]. VABS is a semi-structured parent/caregiver interview assessing communication, daily living skills, socialization and motor skills [22]. Lower scores on the MSEL and VABS indicate greater impairment.

Final study groups were as follow: ASD (*n* = 50) was defined using criteria (based on ADOS and ADI-R) defined by Risi et al. [23]; DD (*n* = 27) included children with MSEL and/or VABS composite score < 70 and an SCQ score below 15; and typically developing (TD, *n* = 68) included children from the general population with no previous diagnosis of ASD or DD, composite scores ≥ 70 on both the MSEL and VABS, and an SCQ score below 15.

Parents also completed the Aberrant Behavior Checklist (ABC) developed to rate inappropriate and maladaptive behaviors [24]. Five subscales have been published for the ABC: irritability, lethargy, stereotypy, hyperactivity and inappropriate speech. Because of the high prevalence of attention deficit hyperactivity disorder (ADHD) symptoms reported in children with ASD [25], we focused on the hyperactivity subscale. We separated this subscale into two subdomains: hyperactivity/impulsivity and inattention. For the hyperactivity/impulsivity subdomain we summed 10 hyperactivity subscale items for a total score range of 0–30; for the inattention subdomain we summed 3 hyperactivity subscale items, for a total score range of 0–9 (See Additional file 1: Table S1, for a detailed list of these items).

### Exposure assessment

We used a high volume small surface sampler (HVS3, CS3 Industries, OR) to vacuum an approximate 5' by 5' area of carpets or rugs located in a main living area. The HVS3 maintains uniform sampling conditions by measuring and controlling the air flow and the pressure drop across a 5-inch nozzle. After collection, dust was stored at −20 °C. Prior to analyses, the dust was sieved to 105 μm with a stainless steel sieve. All samples were spiked with 400 ng of surrogate recovery standard (diphenyl isophthalate) and extracted with dichloromenthane using Soxhlet extraction apparatus. Extracts were concentrated to 5 mL and analyzed using gas chromatograph-mass spectrometer (GS-MS) equipped with glass capillary (DB-5MS; 30 m) for dimethyl phthalate (DMP), DEP, dibutyl phthalate (DBP), BBzP and DEHP [26].

Information regarding the type of flooring in each room of the study participant’s home (area rug, carpet, wood, wood laminate, ceramic tile, vinyl, natural stone, concrete, other) was collected by study staff during the home visit when the dust was sampled.

### Statistical analyses

For phthalates with a frequency of detection > 90 %, we replaced phthalate concentrations below the limit of detection (LOD) with the lowest concentration detected in our study population divided by square root of 2. When the frequency of detection was < 90 % (i.e., DMP) we used multiple imputation to replace the < LOD values [27].

Many ASD and DD children received the minimum score of 20 on one or more MSEL T-scores, leading to highly left-skewed distributions. To resolve the floor-effect of the score distributions, we converted the raw scores to developmental quotients (DQ) computed as follow: DQ = (age equivalent score/chronological age) × 100 [28].

For each phthalate concentration, we fit multinomial logistic regression models predicting the odds of ASD and the odds of DD, each relative to TD. Similar models were used to examine the association between the presence of vinyl flooring in the home and child’s diagnosis. Associations between each phthalate concentration and each of the outcomes of MSEL-DQ and VABS scores were examined with adjusted linear regression models, stratified for the CHARGE diagnosis. We used negative binomial regressions stratified for the CHARGE diagnosis to study the associations between each phthalate concentrations and the ABC hyperactivity/impulsivity and inattention subdomains. The distributions of MSEL-DQ, VABS and ABC scores were similar in magnitude for ASD and DD cases (Additional file 1: Table S2); hence these two groups were combined for analyses of this continuous variables.

A study using NHANES data has shown temporal trends in urine phthalate concentrations; concentrations of DBP, BBzP, DEP and DEHP metabolites have declined between 2001 and 2010, while diisononyl (DiNP) and diisobutyl (DiBP) metabolite concentrations, which might have been used as substitutes for the other phthalates, have increased [4]. In our study population dust concentrations of BBzP and DEP decreased with increasing (later) dates of collection (Additional file 1: Figure S1). In addition, we observed that ASD cases tended to have their dust collected earlier than TD controls (94 % of the ASD children had dust collected in 2010 compared to 78 % in the TD group). For these reasons, the date of dust collection was included as a covariate in our models. Final models were adjusted for maternal education (bachelor degree or higher; >1 year of college; ≤ high school diploma or <1 year of college or technical/vocational school), child race (white, non-white), child age at assessment (in months), child sex, and date of dust collection (continuous). Candidate confounders were selected *a priori*.

Phthalate concentrations were ln-transformed and the effect estimates and Odds Ratios are reported for a one-unit increase in the ln-transformed phthalate concentrations (μg/g of dust).

### Additional analyses

Boys are more likely to be affected with ASD than girls (sex ratio ~ 4:1) and sex-specific effects on several developmental domains have been previously reported for phthalates [29, 30]. For these reasons, we performed additional analyses restricted to boys. Population size was too small to study associations among girls only (*n* = 30).

## Results

### Dust study population

Compared to CHARGE participants enrolled between 2003 (beginning of the CHARGE study) and July 2011 (end of the dust study), children enrolled in the dust study were more likely to be TD, white non-Hispanic and to have older parents. Maternal education levels, proportion of parents who owned their home or had a delivery paid by a private insurance were higher among the dust study participants compared with the full CHARGE study participants, suggesting higher socio-economic levels (Additional file 1: Table S3).

Among the dust study participants, children with ASD were more likely to be Hispanic and to have older parents than TD children (Table 1). Children with DD were more likely to be Hispanic and female than were TD children, who were frequency-matched on sex distribution to ASD cases. Maternal education was substantially lower in the DD group compared with the TD group (37 % versus 61 % of the mothers had a bachelor degree). Average birth weight was lower in the DD group (2991 g) compared to the ASD and TD group (≥3400 g). Both ASD and DD groups were reported to have greater hyperactivity and inattention problems, as compared with TD children (Additional file 1: Table S1).

**Table 1:** Characteristics of the study sample according to diagnostic group (CHARGE study, *n* = 145)

Characteristics	ASD (*n* = 50)		DD (*n* = 27)		TD (*n* = 68)		*p*-values (ASD versus TD)^a^	*p*-values (DD versus TD)^a^
	N	%	N	%	N	%		
Child gender							0.45	0.02
Male	40	80	17	63	58	85		
Female	10	20	10	37	10	15		
Child race							0.12	0.05
White, non-Hispanic	26	52	14	52	45	66		
Hispanic (any)	17	34	11	41	12	18		
Other	7	14	2	7	11	16		
Child age at dust collection (years)							0.03	0.12
<3	5	10	2	7	11	16		
3 to 4	10	20	6	22	26	38		
>4	35	70	18	67	31	46		
Missing	0	0	1	4	0	0		
Maternal education							0.42	0.04
High school diploma or lower, < 1 year college, technical vocational school	8	16	7	26	6	9		
>1 year college, associate degree	12	24	10	37	21	31		
Bachelor degree	20	40	7	26	29	43		
Graduate school	10	20	3	11	12	18		
Delivery payer							0.76	0.20
Government program/no insurance	5	10	6	22	8	12		
Private insurance	45	90	21	78	60	88		
Regional center catchment area at enrollment							0.88	0.50
Alta, Far Northern and Redwood Coast	26	52	20	74	39	57		
North Bay	9	18	3	11	13	19		
East Bay, San Andreas, and Golden Gate	6	12	2	7	7	10		
Valley Mt, Central Valley, and Kern	9	18	2	7	9	13		
Number of rooms with vinyl flooring							0.16	0.09
0	37	74	11	41	42	62		
≥1	13	26	15	56	26	38		
Missing	0	0	1	4	0	0		
Years of dust collection							0.02	0.38
2010	47	94	18	67	53	78		
2011	3	6	8	30	15	22		
Missing	0	0	1	4	0	0		
Continuous variables	Mean	SD	Mean	SD	Mean	SD		
Paternal age (years)	37	5.1	35	7	34	6	0.01	0.49
Maternal age (years)	34	4.7	33	6	32	6	0.06	0.78
Birth weight (g)	3456	715	2991	771	3402	557	0.48	0.01
Gestational duration (wks)	39	2.1	38	3	39	2	0.54	0.91

*ASD* autism spectrum disorders, *DD* developmental delay, *TD* typically developing

^a^Chi-square test for categorical variables and Mann–Whitney test for continuous variables

### Phthalate concentrations in dust

**Fig. 1:**
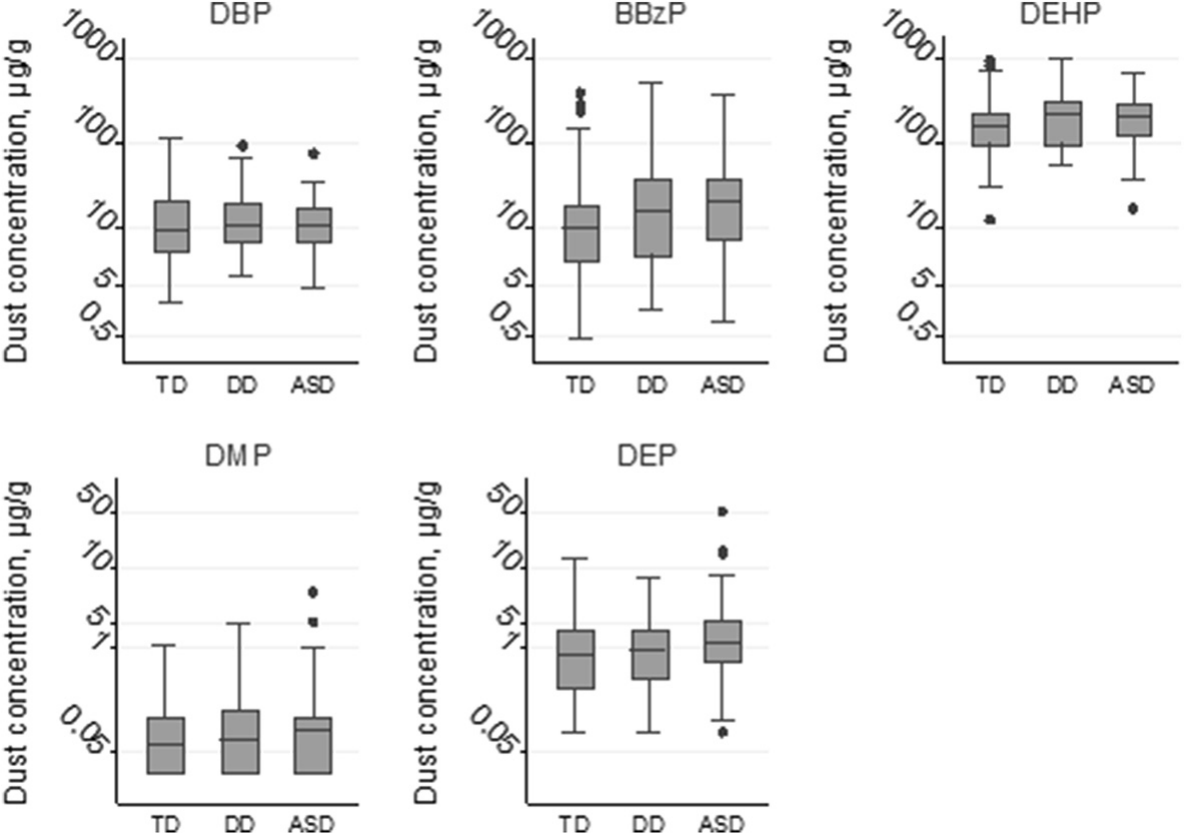
Dust phthalate concentrations (ln-transformed) according to diagnostic group (CHARGE study, *n* = 145). Abbreviation: ASD: autism spectrum disorders, BBzP: butylbenzyl phthalate, DBP: dibutyl phthalate, DD: developmental delay, DEHP: di(2-ethylhexyl) phthalate, DEP: diethyl phthalate, DMP: dimethyl phthalate, TD: typically developing

Detection frequency of phthalates in home dust was 63 % for DMP, 92 % for DEP and 99 % for DBP, BBzP and DEHP. DEHP was the most abundant phthalate measured in house dust, followed by BBzP, DBP, DEP and DMP (Fig. 1, Additional file 1: Table S4). After adjustment for date of dust collection we observed higher concentrations of BBzP in residences with vinyl flooring compared to those that did not contain this type of flooring (β = 0.48 μg/g, 95 % CI: 0.0, 0.9). Vinyl flooring at home was not clearly associated with dust concentration of the other phthalates (*p*-values ≥ 0.2).

### Dust phthalate concentrations and neurodevelopmental outcomes

After adjusting for maternal education, child’s sex, race, age at assessment and date of dust collection, none of the phthalates measured in dust was clearly associated with ASD (*p*-values ≥ 0.30, Table 2). However, we observed an increased risk of DD with increasing dust concentrations of DEHP (OR: 2.12; 95 % CI: 1.10, 4.09) and BBzP (OR: 1.40; 95 % CI: 0.97, 2.04). No other phthalate was clearly associated with DD (Table 2).

**Table 2:** Multinomial Odds Ratios for autism spectrum disorder and developmental delay in relation to dust phthalate concentrations, CHARGE study, n = 144^a^

Phthalates	ASD versus TD		DD versus TD	
	OR	95 % CI	OR	95 % CI
Boys and Girls (*n* = 144)				
Di-methyl phthalate	1.09	[0.79; 1.50]	1.14	[0.75; 1.72]
Di-ethyl phthalate	1.13	[0.76; 1.66]	1.18	[0.70; 1.98]
Di-butyl phthalate	1.06	[0.65; 1.73]	1.63	[0.92; 2.87]
Butyl benzyl phthalate	1.17	[0.85; 1.61]	1.40	[0.97; 2.04]
Di-(2-ethylhexyl) phthalate	1.33	[0.78; 2.25]	2.12	[1.10; 4.09]
Boy only (*n* = 115)				
Di-methyl phthalate	1.12	[0.79; 1.61]	1.12	[0.67; 1.83]
Di-ethyl phthalate	1.01	[0.66; 1.55]	1.16	[0.65; 2.07]
Di-butyl phthalate	1.27	[0.72; 2.25]	1.38	[0.70; 2.72]
Butyl benzyl phthalate	1.20	[0.84; 1.72]	1.59	[1.00; 2.53]
Di-(2-ethylhexyl) phthalate	1.23	[0.69; 2.19]	2.27	[1.00; 5.11]

Analyses adjusted for child age at assessment, child gender and race, maternal education and date of dust collection

*ASD* autism spectrum disorder, *DD* developmental delay, *TD* typically developing

^a^Date of dust collection was missing for one participant

We did not observe any association between any phthalates and the MSEL domains (*p*-values > 0.1, Table 3). Among TD children, DEP was associated with the VABS communication, daily living and adaptive composite standard scores, which respectively differed by −4.4 (95 % CI: −7.6, −1.2), −3.3 (95 % CI: −6.8, 0.3) and −4.5 (95 % CI: −8.2, −0.7) points in association with an one unit increase in the ln-transformed DEP concentration. We observed similar patterns of associations between DBP and these VABS subscales (*p*-values ranged from 0.04 to 0.10, Table 4). DEHP, BBzP and DMP were not clearly associated with any of the VABS domains (Table 3).

**Table 3:** Adjusted associations between dust phthalate concentrations and the MSEL, and VABS scores (CHARGE study, n = 144^a^)

	Dimethyl phthalate		Diethyl phthalate		Dibutyl phthalate		Butylbenzyl phthalate		Di(2-ethylhexyl) phthalate	
	β	95% CI	β	95% CI	β	95% CI	β	95% CI	β	95% CI
ASD and DD children (*n* = 77)										
Mullen (DQ scores)										
Composite score	0.60	[−3.3; 4.5]	0.97	[−3.8; 5.7]	0.13	[−6.1; 6.4]	−2.20	[−6.1; 1.7]	0.30	[−6.2; 6.8]
Expressive language	0.22	[−4.3; 4.8]	1.44	[−4.2; 7.1]	−0.11	[−7.5; 7.3]	−2.90	[−7.5; 1.7]	−1.52	[−9.2; 6.1]
Fine motor	0.76	[−2.5; 4.0]	0.02	[−4.1; 4.1]	−2.60	[−8.0; 2.8]	−1.70	[−5.1; 1.7]	0.64	[−5.0; 6.2]
Receptive language	1.41	[−3.2; 6.0]	1.09	[−4.6; 6.8]	−0.37	[−7.8; 7.1]	−2.20	[−6.9; 2.5]	1.21	[−6.5; 8.9]
Visual reception	−0.01	[−4.8; 4.8]	1.34	[−4.6; 7.3]	3.61	[−4.2; 11]	−2.00	[−7.0; 3.0]	0.89	[−7.3; 9.0]
Vinland (standard scores)										
Composite score	0.29	[−1.9; 2.5]	0.37	[−2.3; 3.0]	−0.22	[−3.7; 3.3]	−0.40	[−2.6; 1.8]	−2.00	[−5.6; 1.6]
Communication	−0.43	[−2.8; 1.9]	−0.20	[−3.1; 2.7]	−1.16	[−4.9; 2.6]	−0.53	[−2.9; 1.9]	−2.46	[−6.3; 1.4]
Daily living skills	1.75	[−0.5; 3.9]	1.53	[−1.3; 4.3]	0.74	[−3.0; 4.5]	0.41	[−1.9; 2.8]	0.01	[−3.8; 3.9]
Motor skills	0.14	[−3.0; 3.3]	2.08	[−1.9; 6.0]	−2.25	[−7.4; 2.9]	−1.34	[−4.6; 2.0]	−4.40	[−9.7; 0.9]
Socialization	0.18	[−2.4; 2.8]	−0.72	[−3.9; 2.5]	2.25	[−1.9; 6.4]	−0.63	[−3.3; 2.0]	−0.32	[−4.7; 4.0]
TD children (*n* = 68)										
Mullen (DQ scores)										
Composite score	0.83	[−2.2; 3.9]	−1.83	[−5.2; 1.5]	−0.38	[−4.0; 3.3]	0.96	[−1.5; 3.4]	1.78	[−2.3; 5.9]
Expressive language	−0.28	[−4.2; 3.6]	−2.17	[−6.8; 2.5]	0.00	[−5.0; 5.0]	0.13	[−3.3; 3.5]	0.71	[−4.9; 6.4]
Fine motor	1.90	[−1.3; 5.1]	1.06	[−2.5; 4.7]	1.01	[−2.9; 4.9]	1.05	[−1.6; 3.7]	3.48	[−0.8; 7.8]
Receptive language	0.63	[−3.4; 4.6]	−2.70	[−7.3; 1.8]	−0.64	[−5.6; 4.3]	0.98	[−2.4; 4.3]	1.41	[−4.2; 7.0]
Visual reception	1.04	[−3.4; 5.5]	−3.50	[−8.2; 1.2]	−1.90	[−7.0; 3.3]	1.67	[−1.8; 5.1]	1.52	[−4.3; 7.4]
Vinland (standard score)										
Composite score	1.19	[−2.4; 4.8]	−4.45	[−8.2; −0.7]	−3.56	[−7.6; 0.5]	0.43	[−2.4; 3.3]	−0.08	[−4.8; 4.6]
Communication	0.39	[−2.7; 3.5]	−4.38	[−7.6; −1.2]	−3.77	[−7.3; −0.3]	−0.46	[−2.9; 2.0]	−1.29	[−5.4; 2.8]
Daily living skills	1.43	[−1.8; 4.6]	−3.29	[−6.8; 0.3]	−3.16	[−7.0; 0.7]	0.14	[−2.5; 2.8]	−1.37	[−5.8; 3.0]
Motor skills	1.56	[−1.8; 4.9]	−2.06	[−5.6; 1.5]	−1.71	[−5.6; 2.2]	0.58	[−2.0; 3.2]	1.36	[−3.0; 5.8]
Socialization	0.15	[−2.8; 3.1]	−2.90	[−6.0; 0.3]	−1.36	[−4.8; 2.1]	1.28	[−1.1; 3.6]	1.24	[−2.7; 5.2]

Analyses adjusted for child age at assessment, child gender and race, maternal education and date of dust collection

*ASD* autism spectrum disorder, *DD* developmental delay, *TD* typically developing

^a^Date of dust collection was missing for one participant

**Table 4:** Incidence rate ratios for ABC scores among DD and ASD in relation to dust phthalate concentrations, CHARGE study

	Hyperactivity/ Impulsivity		Inattention	
	IRR	95% CI	IRR	95% CI
Boys and Girls				
Dimethyl phthalate	0.97	[0.82; 1.15]	1.02	[0.91; 1.14]
Diethyl phthalate	1.16	[0.95; 1.42]	1.06	[0.95; 1.19]
Dibutyl phthalate	1.35	[0.98; 1.85]	1.13	[0.91; 1.39]
Butylbenzyl phthalate	1.02	[0.86; 1.21]	1.03	[0.90; 1.18]
Di(2-ethylhexyl) phthalate	0.80	[0.61; 1.06]	1.03	[0.88; 1.20]
Boys only				
Dimethyl phthalate	0.98	[0.82; 1.17]	1.01	[0.90; 1.12]
Diethyl phthalate	1.25	[1.02; 1.54]	1.13	[1.03; 1.24]
Dibutyl phthalate	1.63	[1.22; 2.19]	1.25	[0.96; 1.62]
Butylbenzyl phthalate	1.10	[0.88; 1.37]	1.03	[0.91; 1.17]
Di(2-ethylhexyl) phthalate	0.94	[0.68; 1.30]	1.15	[0.95; 1.39]

Analyses adjusted for child age at assessment, child gender and race, maternal education and date of dust collection

Associations between phthalates and ABC subscales are not reported among TD because of the high prevalence of 0 score in this group (~50 %)

*IRR* Incidence rate ratio

After excluding girls, DBP and DEP were associated with greater hyperactivity/impulsivity and inattention scores among DD and ASD children (ABC subdomains, Table 4).

### Vinyl-flooring and risk of ASD and DD during childhood

Children with DD (56 %) were more likely to live in a home with at least one room that had vinyl flooring compared to TD (38 %) and ASD (26 %) children (Table 1). After adjusting for maternal education, child’s sex, age and race, we did not observe any association between the presence of vinyl flooring at home and ASD (OR: 0.55; 95 % CI: 0.24, 1.27) or DD (OR: 1.75; 95 % CI: 0.65, 4.70).

P-values and confidence intervals reported in the manuscript are not corrected for multiple comparisons. In additional analysis, we applied the false discovery rate correction to adjust for multiple testing; none of the associations reported in the manuscript remained significant.

## Discussion

In our study population, whereas no phthalate was clearly associated with ASD, dust concentrations of DEHP and BBzP were associated with an increased risk of DD. Among TD children, DEP and DBP dust concentrations were associated with impairments in several adaptive skills assessed with VABS. Among ASD and DD boys, these same compounds, DEP and DBP, were associated with more hyperactivity/impulsivity and inattention problems. DEHP and BBzP are high molecular weight phthlates used in PVC plastics, while DEP and DBP are low molecular weight phthalates used among other in personal care products and as coatings in pharmaceutical industry [5].

### Phthalate concentrations in house dust

Several studies have measured phthalate concentrations in indoor dust (Additional file 1: Table S6). In all of these studies, DEHP was the most abundant phthalate measured in dust; its concentration was 10 to 100 times higher than any other phthalates. That might be explained by its extensive use and its low vapor pressure [31, 32]. Compared with previous studies measuring phthalates in indoor dust collected in U.S. residences, median concentrations were lower in our population (Additional file 1: Table S6). Medians were 187, 13, 10, 1 and 0.07 μg/g for DEHP, BBzP, DBP, DEP and DMP in our study population, compared to medians of 304–386, 21–45, 13–20, 2–5 and 0.08 μg/g, respectively, in previous studies [7, 26, 33]. Discrepancies in phthalate levels across studies might be explained by differences in the dates of dust collection (depending of the studies, dust were collected in 1999–2001 [7], 2004 [26], 2007–2008 and 2010 [33] and 2010–2011 (our study)) and by the use of varying methods for dust collection. We only vacuumed a 5′ by 5′ area of rug or carpet while other studies collected dust from the participant’s vacuum cleaner bags [26, 33] or vacuumed multiple surfaces in several rooms of the residence [7].

In our study population, we observed a decrease in BBzP and DEP dust concentrations in association with increasing (later) dates of collection. The method of dust collection and analysis did not change during the study period, reducing the possibility that these decreases were an artefact. Declining phthalate exposures have been previously observed: a 20 to 50 % decline in DEP, DBP, BBzP and DEHP urinary metabolite concentrations was reported between 2001 and 2010 in NHANES [4].

### Phthalates and neurodevelopmental outcomes

#### ASD and DD

Although none of the dust phthalate concentrations was associated with ASD risk, we observed an increased risk of having a child with DD in association with increased concentrations of DEHP and BBzP in house dust. Results of previous studies exploring the associations between phthalate exposures and early mental development assessed using the Mental Development Index (MDI) of the Bayley Scales of Infant Development are mixed. Higher DEHP metabolites measured in maternal urine collected during pregnancy were associated with lower MDI scores among 6 month old Korean boys [34] and in a different study, among 24 to 36 month old Mexican girls [30]. In another population of 3 year-olds from New-York City, no association was reported between DEHP and MDI scores [29]. No association was observed between BBzP metabolite and MDI scores [29, 30].

#### Adaptive functions

Among TD children, higher DEP and DBP dust concentrations were associated with poorer scores in a composite of adaptive behaviors (VABS), which is comprised of the communication, daily living, socialization and motor skills domains. These compounds also showed associations with the communication and daily living domains. For children aged 3 years or older, the communication domain explores receptive (e.g., abilities to follow instructions) and expressive (e.g., name colors) language as well as written abilities (e.g., recognize own name in printed form, identify one or more letter of the alphabet). The daily living domain includes questions regarding the child’s personal behavior (e.g., being toilet trained and able to dress oneself) and involvement in simple household chores and ability to use home devices such as a TV or phone [22]. An association between MEP (DEP metabolite) concentration in maternal urine collected during pregnancy and social communication has been observed in a previous study that used the Social Responsiveness Scale in 137 children aged 7 to 9 years from the general population [12].

#### Hyperactivity / impulsivity

We were not able to assess associations with the hyperactivity/impulsivity and inattention subdomains of the ABC among TD children because of the high prevalence of null scores in this group (~50 %). Among boys with ASD and DD, a higher concentration of DBP and DEP in dust tended to be associated with greater hyperactivity/impulsivity and inattention. None of the other phthalates assessed in our study was associated with these ABC subscales. MBP (urinary metabolite of DBP) and MEP (urinary metabolite of DEP) concentrations measured in maternal urine have been associated with ADHD-like behaviors, including increased aggression (MBP), externalizing problems (MBP and MEP) and attention problems (MEP) among 4 to 9 years old boys from New-York City [35].

#### Vinyl flooring and neurodevelopmental outcomes

We found no association of vinyl flooring with the diagnosis of ASD or DD. Among Swedish children, an increased risk of ASD has been associated with PVC flooring in the parents’ bedroom [15]. We were limited in studying this association because vinyl flooring was found in 37 % of residences in our population compared to the Sweden cohort in which vinyl flooring has been reported in 45 % of the parents’ bedrooms and 52 % of the children’s bedrooms [15]. In addition, we did not have data on the type of the room that had vinyl flooring, which was a limitation.

#### Strengths and limitations

Strengths of this study include the case–control design, which allowed us to study a rare neurodevelopmental condition, namely ASD; confirmation of ASD, DD and TD diagnoses by trained clinicians using gold-standard assessments; and the collection of detailed data on covariates. In order to avoid bias in the chemical analysis, all of the dust samples were shipped to the lab without any information regarding the child’s diagnosis.

Our sample size was small with only 50 cases of ASD, 27 cases of DD and 68 TD children enrolled, and compared to the whole CHARGE population, participants enrolled in the dust substudy had a higher socio-economic status, though were similar on perinatal factors.

Despite frequent vacuuming, indoor dust has a low turn-over (on the order of years) and indoor models have predicted long half-lives in this media for many semi volatile compounds [36]. However, data regarding the temporal reproducibility of indoor dust phthalate concentrations over long periods of time are needed to better understand the extent to which phthalate concentrations in house dust collected 2 to 5 years after birth could be a good proxy of exposure during pregnancy and the first years of life, two crucial time points for brain development. In addition, because symptoms of ASD and DD occurred before the dust collection, we cannot rule out reverse causality. Having a child with ASD or DD might change behaviors affecting the dust phthalate concentrations (e.g., the frequency of house cleaning). Another limitation of using dust concentrations to assess exposure is that we missed other sources which might account for a substantial contribution to exposure, depending on the specific phthalate [37]. Finally, the set of phthalates we measured in dust did not include diisononyl phthalate and diisodecyl phthalate, two phthalates that have not been extensively studied in relation to child neurodevelopment, despite increased exposures to these chemicals in the general population over the past decade [4]. Finally, we performed many comparisons, and as it is suggested by the fact that none of the associations remained significant after correction for multiple comparisons, our findings might be due to chance.

## Conclusion

We observed an increased risk of DD in association with DEHP and BBzP concentrations in house dust; poorer adaptive function in association with DEP and DBP; and a trend towards greater hyperactivity-impulsivity among boys with ASD or DD in association with DBP and DEP. ASD risk was not associated with any of the measured phthalates in house dust. Taken together with results of previous studies [16], these findings raise concerns that phthalate exposure may adversely affect several domains of child neurodevelopment. The use of house dust collected between ages 2 and 5 years precluded distinguishing recent exposures from earlier ones (e.g., during gestation). These measurements also likely underestimated total exposures by missing the contributions from other sources, such as alimentation. As the sample size was small, we also cannot exclude that some of our findings may be due to chance. Hence, both positive and null findings should be interpreted cautiously. Replications are needed in other populations with a large sample size and improved exposure assessment. Biomarkers measured in maternal urine collected during pregnancy and in child urine are likely to give a more relevant estimate of phthalate exposure than dust concentration, but the low ICCs reported for phthalates in urine collected during pregnancy and childhood [17, 38]) suggest that repeated measurements would be needed.

## Additional file

Additional file 1: 
**Table S1.** Aberrant Behavior Checklist hyperactivity subscale items used to compute the hyperactivity/impulsivity subdomain and the inattention subdomain. **Table S2.** MSEL, VABS and ABC scores according to diagnostic groups (CHARGE study, n = 145). **Table S3.** Characteristics of the dust study population and comparison with the whole CHARGE case-control study enrolled before the end of the dust study (2003 – 2011). **Table S4.** Phthalate concentrations in house dust (μg/g of dust, CHARGE study, n = 145). **Table S5.** Adjusted associations between dust phthalate concentrations and the MSEL and VABS scores among boys (CHARGE study, n = 115). **Table S6.** Comparison of phthalate concentrations measured in house dust in different studies. **Figure S1.** Dust phthalate concentrations (μg/g) according to the date of dust collection.

## Abbreviations

ABC: Aberrant behavior checklist
ASD: Autism spectrum disorder
BBzP: Butylbenzyl phthalate
DBP: Dibutyl phthalate
DD: Developmental delay
DEP: Diethyl phthalate
DEHP: Di(2-ethylhexyl) phthalate
DMP: Dimethyl phthalate
ICC: Intraclass correlation coefficient
MEP: Monoethyl phthalate
MSEL: Mullen Scales of Early Learning
PVC: Polyvinyl chloride
SCQ: Social communication questionnaire
TD: Typically developed
VABS: Vineland Adaptive Behavior Scales
