# Supplementary material for: Phthalate concentrations in house dust in relation to autism spectrum disorder and developmental delay in the CHildhood Autism Risks from Genetics and the Environment (CHARGE) study
Source: Environ Health. 2015 Jun 26;14:56. doi: 10.1186/s12940-015-0024-9 (PMC4507319; doi:10.1186/s12940-015-0024-9)
Supplement: Additional file 1: — Table S1. Aberrant Behavior Checklist hyperactivity subscale items used to compute the hyperactivity/impulsivity subdomain and the inattention subdomain. Table S2. MSEL, VABS and ABC scores according to diagnostic groups (CHARGE study, n = 145). Table S3. Characteristics of the dust study population and comparison with the whole CHARGE case-control study enrolled before the end of the dust study (2003 – 2011). Table S4. Phthalate concentrations in house dust (μg/g of dust, CHARGE study, n = 145). Table S5. Adjusted associations between dust phthalate concentrations and the MSEL and VABS scores among boys (CHARGE study, n = 115). Table S6. Comparison of phthalate concentrations measured in house dust in different studies. Figure S1. Dust phthalate concentrations (μg/g) according to the date of dust collection. [file 12940_2015_24_MOESM1_ESM.pdf]

**SUPPLEMENTAL MATERIAL**

# Phthalate concentrations in house dust in relation to autism spectrum disorder and developmental delay in the CHildhood Autism Risks from Genetics and the Environment (CHARGE) Study.

Claire Philippat, Deborah Bennett, Paula Krakowiak, Melissa Rose, Hyun-Min Hwang, Irva Hertz-Picciotto

## Table of contents

Table 1: Aberrant Behavior Checklist hyperactivity subscale items used to compute the hyperactivity/impulsivity subdomain and the inattention subdomain ........................................... 2

Table 2: MSEL, VABS and ABC scores according to diagnostic groups (CHARGE study, n = 145) ........................................................................................................................ 3

Table 3: Characteristics of the dust study population and comparison with the whole CHARGE case-control study enrolled before the end of the dust study (2003 – 2011). ................................ 4

Table 4: Phthalate concentrations in house dust (µg/g of dust, CHARGE study, n = 145)........... 5

Table 5: Adjusted associations between dust phthalate concentrations and the MSEL and VABS scores among boys (CHARGE study, n = 115)................................................................. 6

Table 6: Comparison of phthalate concentrations measured in house dust in different studies..... 7

Figure 1: Dust phthalate concentrations (µg/g) according to the date of dust collection .............. 8

Table 1: Aberrant Behavior Checklist hyperactivity subscale items used to compute the hyperactivity/impulsivity subdomain and the inattention subdomain

| Subdomain | Subscale items used |
|---|---|
| Hyperactivity/Impulsivity | Hyperactivity subscale items 1, 7, 13, 15, 21, 31, 38, 39, 48, and 54:<br>1. Excessively active at home, school, work, or elsewhere<br>7. Boisterous (inappropriately noisy and rough)<br>13. Impulsive (acts without thinking)<br>15. Restless, unable to sit still<br>21. Disturbs others<br>31. Disrupts group activities<br>38. Does not stay in seat (e.g., during lesson or training periods, meals, etc.)<br>39. Will not sit still for any length of time<br>48. Constantly jumps or runs around the room<br>54. Tends to be excessively active |
| Inattention | Hyperactivity subscale items 28,44,51<br>28. Does not pay attention to instructions<br>44. Easily distractible<br>51. Pays no attention when spoken to |

Table 2: MSEL, VABS and ABC scores according to diagnostic groups (CHARGE study, n = 145)

| | TD | | | | DD | | | | ASD | | | |
|---|---|---|---|---|---|---|---|---|---|---|---|---|
| | N | Percentiles | | | N | Percentiles | | | N | Percentiles | | |
| | | 5 | 50 | 95 | | 5 | 50 | 95 | | 5 | 50 | 95 |
| **Vineland Adaptive Behavior Scales (standard scores)** | | | | | | | | | | | | |
| Communication | 68 | 80 | 103 | 126 | 27 | 49 | 67 | 82 | 50 | 52 | 69 | 94 |
| Daily living skills | 68 | 72 | 93 | 117 | 27 | 51 | 64 | 86 | 50 | 54 | 68 | 93 |
| Socialization | 68 | 85 | 104 | 124 | 27 | 55 | 73 | 107 | 50 | 53 | 67 | 88 |
| Motor skills | 68 | 83 | 107 | 130 | 27 | 49 | 60 | 85 | 50 | 54 | 77 | 107 |
| Adaptive behavior Composite score | 68 | 83 | 98 | 127 | 27 | 47 | 61 | 87 | 50 | 53 | 65 | 88 |
| **Mullen (DQ scores)** | | | | | | | | | | | | |
| Visual Reception | 68 | 88 | 114 | 150 | 27 | 27 | 60 | 103 | 50 | 40 | 70 | 120 |
| Fine Motor | 68 | 78 | 103 | 127 | 27 | 33 | 58 | 78 | 50 | 43 | 67 | 106 |
| Receptive Language | 68 | 80 | 112 | 142 | 27 | 18 | 56 | 81 | 50 | 25 | 52 | 109 |
| Expressive Language | 68 | 79 | 105 | 139 | 27 | 18 | 52 | 81 | 50 | 23 | 61 | 109 |
| Composite score | 68 | 88 | 112 | 131 | 27 | 26 | 60 | 83 | 50 | 37 | 61 | 102 |
| **Aberrant Behavior Checklist (hyperactivity subscales)** | | | | | | | | | | | | |
| Hyperactivity / impulsivity | 64 | 0 | 1 | 7 | 22 | 0 | 4 | 20 | 48 | 0 | 7 | 20 |
| Inattention | 64 | 0 | 0 | 2 | 24 | 0 | 1 | 6 | 49 | 0 | 3 | 6 |

Abbreviation: ASD: autism spectrum disorder, CI: confidence interval, DD: developmental delay, TD: typically developing

Table 3: Characteristics of the dust study population and comparison with the whole CHARGE case-control study enrolled before the end of the dust study (2003 – 2011).

| **Characteristics** | **CHARGE participant enrolled in the dust study (n = 145)** | | **CHARGE participant enrolled until the end of the dust study (n = 1092)** | | **P-values[a]** |
|---|---|---|---|---|---|
| | **N** | **%** | **N** | **%** | |
| **Child diagnosis** | | | | | 0.001 |
| TD | 68 | 47 | 304 | 32 | |
| DD | 27 | 19 | 188 | 20 | |
| ASD | 50 | 34 | 455 | 48 | |
| **Child gender** | | | | | 0.61 |
| Male | 115 | 79 | 768 | 81 | |
| Female | 30 | 21 | 179 | 19 | |
| **Child race** | | | | | 0.09 |
| White, Non-Hispanic | 85 | 59 | 461 | 49 | |
| Hispanic (any) | 40 | 28 | 307 | 32 | |
| other | 20 | 14 | 175 | 18 | |
| missing | 0 | 0 | 4 | 0 | |
| **Maternal education** | | | | | |
| High school diploma and lower, < 1 year college, technical vocational school | 21 | 14 | 265 | 28 | 0.002 |
| > 1year college, associate degree | 43 | 30 | 285 | 30 | |
| Bachelor degree | 56 | 38 | 280 | 30 | |
| Graduate school | 25 | 17 | 116 | 12 | |
| missing | 0 | 0 | 1 | 0 | |
| **Delivery payer** | | | | | 0.02 |
| Government program / no Insurance | 19 | 13 | 202 | 21 | |
| Private insurance | 126 | 87 | 743 | 78 | |
| Missing | 0 | 0 | 2 | 0 | |
| **Own their home** | | | | | |
| Yes | 111 | 76 | 590 | 62 | 0.002 |
| No | 27 | 19 | 290 | 31 | |
| missing | 7 | 5 | 67 | 7 | |
| **Regional center catchment area at enrollment** | | | | | 0.03 |
| Alta, Far Northern, and Redwood Coast | 85 | 59 | 459 | 48 | |
| North Bay | 25 | 17 | 143 | 15 | |
| East Bay, San Andreas, and Golden Gate | 15 | 10 | 121 | 13 | |
| Valley Mt, Central Valley, and Kern | 20 | 14 | 224 | 24 | |
| | **Mean** | **SD** | **Mean** | **SD** | |
| **Paternal age** | 35 | 6 | 33 | 7 | <0.001 |
| **Maternal age** | 33 | 6 | 30 | 6 | <0.001 |
| **Birth weight** | 3344 | 674 | 3407 | 687 | 0.25 |
| **Gestational duration** | 39 | 2 | 39 | 2 | 0.21 |

[a] Chi-square test for categorical variables and Mann-Whitney test for continuous variables.

Table 4: Phthalate concentrations in house dust (µg/g of dust, CHARGE study, n = 145)

| | **% > LOD** | **Percentiles** | | | | |
|---|---|---|---|---|---|---|
| | | **5** | **25** | **50** | **75** | **95** |
| Dimethyl phthalate | 63 | < LOD | <LOD | 0.07 | 0.14 | 0.53 |
| Diethyl phthalate | 92 | < LOD | 0.41 | 1.01 | 1.75 | 6.67 |
| Dibutyl phthalate | 99 | 2.71 | 5.91 | 10.3 | 17.9 | 36.8 |
| Butylbenzyl phthalate | 99 | 1.07 | 5.24 | 13.4 | 34.1 | 200 |
| Di(2-ethylhexyl) phthalate | 99 | 40.4 | 103 | 187 | 301 | 656 |

Table 5: Adjusted associations between dust phthalate concentrations and the MSEL and VABS scores among boys (CHARGE study, n = 115)

| | Dimethyl phthalate | | Diethyl phthalate | | Dibutyl phthalate | | Butylbenzyl phthalate | | Di(2-ethylhexyl) phthalate | |
|---|---|---|---|---|---|---|---|---|---|---|
| | **β** | **95%CI** | **β** | **95%CI** | **β** | **95%CI** | **β** | **95%CI** | **β** | **95%CI** |
| **ASD and DD children (n = 57)** | | | | | | | | | | |
| **Mullen (DQ scores)** | | | | | | | | | | |
| Composite score | -0.89 | [-5.2 ; 3.4] | -0.77 | [-6.1 ; 4.5] | 1.10 | [-7.0 ; 9.2] | -1.07 | [-5.7 ; 3.6] | 2.00 | [-5.8 ; 9.8] |
| Expressive language | -1.67 | [-6.8 ; 3.5] | 0.80 | [-5.6 ; 7.2] | 1.20 | [-8.5 ; 10.9] | -0.87 | [-6.5 ; 4.7] | -0.46 | [-9.8 ; 8.9] |
| Fine motor | -0.57 | [-4.3 ; 3.1] | -3.08 | [-7.7 ; 1.5] | -3.02 | [-10.1 ; 4.0] | -1.86 | [-5.9 ; 2.2] | 1.78 | [-5.0 ; 8.6] |
| Receptive language | 0.86 | [-4.3 ; 6.1] | 0.68 | [-5.7 ; 7.1] | 2.48 | [-7.2 ; 12.2] | 0.28 | [-5.3 ; 5.9] | 3.46 | [-5.9 ; 12.8] |
| Visual reception | -2.17 | [-7.7 ; 3.3] | -1.49 | [-8.4 ; 5.4] | 3.75 | [-6.7 ; 14.2] | -1.85 | [-7.9 ; 4.2] | 3.22 | [-6.9 ; 13.3] |
| **Vineland (standard scores)** | | | | | | | | | | |
| Composite score | 0.00 | [-2.4 ; 2.4] | -1.49 | [-4.5 ; 1.5] | -0.78 | [-5.3 ; 3.8] | -0.19 | [-2.8 ; 2.5] | -2.34 | [-6.7 ; 2.0] |
| Communication | -0.87 | [-3.5 ; 1.8] | -1.82 | [-5.1 ; 1.4] | -1.74 | [-6.7 ; 3.3] | 0.28 | [-2.6 ; 3.2] | -2.30 | [-7.1 ; 2.5] |
| Composite score | 0.00 | [-2.4 ; 2.4] | -1.49 | [-4.5 ; 1.5] | -0.78 | [-5.3 ; 3.8] | -0.19 | [-2.8 ; 2.5] | -2.34 | [-6.7 ; 2.0] |
| Daily living skills | 1.09 | [-1.0 ; 3.2] | -0.41 | [-3.1 ; 2.3] | -0.35 | [-4.5 ; 3.8] | 0.15 | [-2.2 ; 2.5] | -0.98 | [-5.0 ; 3.0] |
| Motor skills | 0.69 | [-2.7 ; 4.1] | -0.89 | [-5.2 ; 3.4] | -1.65 | [-8.2 ; 4.9] | -1.62 | [-5.4 ; 2.1] | -3.28 | [-9.6 ; 3.0] |
| Socialization | -0.48 | [-3.5 ; 2.5] | -1.34 | [-5.1 ; 2.4] | 0.68 | [-5.0 ; 6.4] | 0.20 | [-3.1 ; 3.5] | -0.90 | [-6.4 ; 4.6] |
| **TD children (n =58)** | | | | | | | | | | |
| **Mullen (DQ scores)** | | | | | | | | | | |
| Composite score | 0.21 | [-3.2 ; 3.6] | -1.54 | [-5.1 ; 2.0] | 0.20 | [-3.6 ; 4.0] | 1.23 | [-1.6 ; 4.0] | 1.29 | [-3.1 ; 5.6] |
| Expressive language | -0.57 | [-5.0 ; 3.8] | -1.83 | [-6.7 ; 3.0] | 0.32 | [-5.0 ; 5.6] | 1.08 | [-2.8 ; 5.0] | 0.75 | [-5.3 ; 6.7] |
| Fine motor | 1.00 | [-2.4 ; 4.4] | 0.78 | [-2.8 ; 4.4] | 2.10 | [-1.8 ; 6.0] | 0.09 | [-2.8 ; 3.0] | 2.31 | [-2.1 ; 6.7] |
| Receptive language | -0.24 | [-4.6 ; 4.2] | -1.92 | [-6.7 ; 2.8] | 0.03 | [-5.1 ; 5.2] | 1.36 | [-2.4 ; 5.1] | 0.83 | [-5.0 ; 6.7] |
| Visual reception | 0.65 | [-4.3 ; 5.7] | -3.17 | [-8.3 ; 1.9] | -1.66 | [-7.2 ; 3.9] | 2.37 | [-1.7 ; 6.4] | 1.25 | [-5.1 ; 7.6] |
| **Vineland (standard scores)** | | | | | | | | | | |
| Composite score | 1.28 | [-2.7 ; 5.3] | -4.30 | [-8.1 ; -0.5] | -3.39 | [-7.6 ; 0.8] | 0.98 | [-2.1 ; 4.1] | -0.80 | [-5.7 ; 4.0] |
| Communication | 0.87 | [-2.6 ; 4.3] | -3.66 | [-7.1 ; -0.3] | -4.05 | [-7.7 ; -0.4] | 0.45 | [-2.4 ; 3.3] | -1.38 | [-5.7 ; 3.0] |
| Composite score | 1.28 | [-2.7 ; 5.3] | -4.30 | [-8.1 ; -0.5] | -3.39 | [-7.6 ; 0.8] | 0.98 | [-2.1 ; 4.1] | -0.80 | [-5.7 ; 4.0] |
| Daily living skills | 1.13 | [-2.4 ; 4.6] | -3.56 | [-7.2 ; 0.1] | -3.01 | [-7.0 ; 0.9] | 0.68 | [-2.3 ; 3.6] | -1.98 | [-6.5 ; 2.6] |
| Motor skills | 1.51 | [-2.0 ; 5.0] | -2.15 | [-5.7 ; 1.4] | -1.46 | [-5.3 ; 2.4] | 0.98 | [-1.8 ; 3.8] | 1.08 | [-3.3 ; 5.5] |
| Socialization | 0.28 | [-3.0 ; 3.6] | -2.90 | [-6.2 ; 0.4] | -1.15 | [-4.8 ; 2.5] | 1.21 | [-1.5 ; 3.9] | 0.38 | [-3.8 ; 4.5] |

Analyses adjusted for child age at assessment, child race, maternal education and date of dust collection.

Abbreviation: ASD: autism spectrum disorder, DD: developmental delay, TD: typically developing

Table 6: Comparison of phthalate concentrations measured in house dust in different studies

| Country and year of dust collection | Concentrations measured in dust (median, ug/g) | | | | |
|---|---|---|---|---|---|
| | DMP | DEP | DBP | BBzP | DEHP |
| USA (CA), 2011-2010 (present study) | 0.07 | 1 | 10 | 13 | 187 |
| Japan, 2009 and 2010 [1] | < LOD | < LOD | 16.6 | 2 | 1110 |
| USA (NY), 2007-2008, 2010 [2] | 0.08 | 2 | 13.1 | 21.1 | 304 |
| China, 2007-2008, 2010 [2] | 0.2 | 0.4 | 20.1 | 0.2 | 228 |
| Denmark, 2008 [3] | / | 1.7 | 15 | 3.7 | 210 |
| USA (CA), 2004 [4] | / | / | / | / | 386 |
| Sweden, 2001-2002 [5] | / | 0 | 45 | 135 | 770 |
| Germany, 2000-2001 [6] | 1.5 | 6.1 | 47 | 29.7 | 703 |
| USA (MA), 1999-2001 [7] | / | 4.98 | 20.1 | 45.4 | 340 |
| Germany [8] | / | / | 87.4 | 15.2 | 480 |

Figure 1: Dust phthalate concentrations (µg/g) according to the date of dust collection

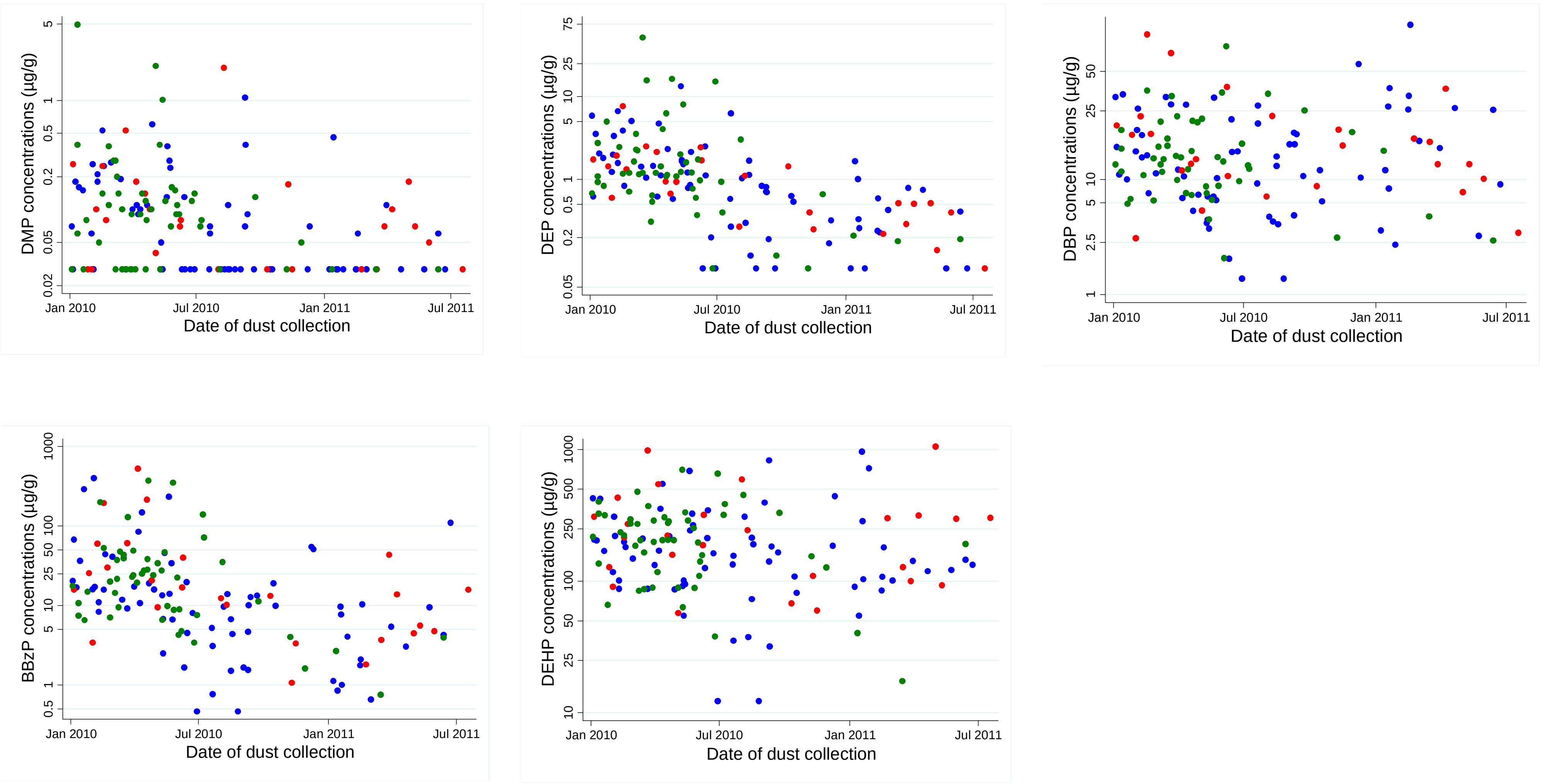


Blue circles represent TD children, red circles DD children and green circles ASD children.
Abbreviations: BBzP: Butylbenzyl phthalate, DBP: Dibutyl phthalate, DEP: Diethyl phthalate, DMP: Dimethyl phthalate, DEHP: Di(2-ethylhexyl) phthalate

## References


1. Ait Bamai Y, Araki A, Kawai T, Tsuboi T, Saito I, Yoshioka E, Kanazawa A, Tajima S, Shi C, Tamakoshi A *et al*: Associations of phthalate concentrations in floor dust and multi-surface dust with the interior materials in Japanese dwellings. *Sci Total Environ* 2014, 468-469:147-157.

2. Guo Y, Kannan K: Comparative assessment of human exposure to phthalate esters from house dust in China and the United States. *Environ Sci Technol* 2011, 45(8):3788-3794.

3. Langer S, Weschler CJ, Fischer A, Bekö G, Toftum J, Clausen G: Phthalate and PAH concentrations in dust collected from Danish homes and daycare centers. *Atmospheric Environment* 2010, 44(19):2294-2301.

4. Hwang HM, Park EK, Young TM, Hammock BD: Occurrence of endocrine-disrupting chemicals in indoor dust. *Sci Total Environ* 2008, 404(1):26-35.

5. Bornehag CG, Lundgren B, Weschler CJ, Sigsgaard T, Hagerhed-Engman L, Sundell J: Phthalates in indoor dust and their association with building characteristics. *Environ Health Perspect* 2005, 113(10):1399-1404.

6. Fromme H, Lahrz T, Piloty M, Gebhart H, Oddoy A, Ruden H: Occurrence of phthalates and musk fragrances in indoor air and dust from apartments and kindergartens in Berlin (Germany). *Indoor Air* 2004, 14(3):188-195.

7. Rudel RA, Camann DE, Spengler JD, Korn LR, Brody JG: Phthalates, alkylphenols, pesticides, polybrominated diphenyl ethers, and other endocrine-disrupting compounds in indoor air and dust. *Environ Sci Technol* 2003, 37(20):4543-4553.

8. Abb M, Heinrich T, Sorkau E, Lorenz W: Phthalates in house dust. *Environ Int* 2009, 35(6):965-970.
